# Complete genome sequence of a methylotroph, *Methylobacillus glycogenes*, isolated from soil

**DOI:** 10.1128/mra.00663-26

**Published:** 2026-09-10

**Authors:** Shogo Nakabori, Tomoya Maeda

**Affiliations:** 1 Laboratory of Microbial Physiology, Research Faculty of Agriculture, Hokkaido University 12810, Sapporo, Japan; 2 RIKEN Center for Biosystems Dynamics Research 208578, Kobe, Hyogo, Japan; Indiana University Bloomington, Bloomington, Indiana, USA

**Keywords:** methylotrophs, *Methylobacillus*

## Abstract

Methanol is a sustainable, low-cost feedstock produced from CO_2_ or non-food biomass. We report the complete genome sequence of *Methylobacillus glycogenes* strain PKM581, a soil-derived obligate methylotroph enriched on methanol. The genome comprises a single circular chromosome of 3,397,625 bp.

## ANNOUNCEMENT

Methanol can be synthesized from CO_2_ or non-food biomass and is a low-cost C1 substrate (1). *Methylobacillus glycogenes* is a gram-negative obligate methylotroph capable of producing amino acids from methanol (2, 3). Strain PKM581 was isolated from soil collected 5 cm below the ground surface in Osaka, Japan (34.7523°N, 135.4963°E). A 1g soil was suspended in 25 mL phosphate-buffered saline and incubated at 25°C and 150 rpm for 24 h. After centrifugation at 1,000 rpm for 1 min, glycerol was added to the supernatant to 10% (vol/vol), and the resulting glycerol stock was stored at −80°C. A 240 μL aliquot was inoculated into 6.5 mL M9 minimal medium supplemented with 1% (vol/vol) nutrient broth and cultured at 25°C and 150 rpm for 7 days. Ten microliters of culture was then transferred to 190 μL M9 minimal medium containing 0.4% (vol/vol) methanol as the sole carbon and energy source in a 96-well microplate and incubated at 25°C and 180 rpm for 7 days. Weekly 10 μL transfers into 190 μL fresh medium were repeated 12 times. PKM581 was isolated from the final glycerol stock by three successive rounds of single-colony isolation on M9 agar containing 0.4% methanol.

For sequencing, PKM581 was grown in M9 minimal medium containing 0.4% methanol at 30°C and 180 rpm for 48 h. Genomic DNA was extracted using the Monarch HMW DNA Extraction Kit (New England Biolabs, MA, USA) according to the manufacturer’s instructions, and aliquots of the same DNA extraction were used to prepare both sequencing libraries. Sequencing was performed by Beijing Genomics Institute (BGI, Shenzhen, China). For DNBSEQ sequencing, 1 µg DNA was fragmented using a Covaris LE220 (Covaris, MA, USA), size selected to 200–400 bp, prepared with the MGIEasy Universal DNA Library Prep Set, and sequenced on a DNBSEQ-G400 (MGI Tech Co., Ltd., Shenzhen, China). SOAPnuke v1.5.6 (-l 20 -q 40% -n 0.1% -d) (4) yielded 8,738,130 reads totaling 1,252 Mb. For Cyclone sequencing, a ligation-based library was prepared without fragmentation or size selection and sequenced on a CycloneSEQ G100-ER using a WuTong sequencing chip (BGI; H930-000001-00). Base calling, barcode demultiplexing, and trimming used wt_basic_v1.5.12; reads with mean quality <7 were automatically removed, and reads <1,000 bp were excluded. The filtered data set comprised 104,426 reads totaling 1,422 Mb (read N50, 26,054 bp).

Long reads were corrected and assembled using Canu v2.2 (useGrid = 0 and corOvlMemory = 4) (5). Circularity was assessed by sequence-loop judgment within the sequencing provider’s assembly workflow. Single-base errors were corrected with DNBSEQ reads using GATK v3.4-0 (-stand_call_conf 20.0, -stand_emit_conf 20.0, and -allowPotentiallyMisencodedQuals) (6). The final chromosome was oriented with nucleotide 1 corresponding to the start codon of *dnaA*. Annotation was performed with DFAST v1.2.0 using default parameters (7). Final DNBSEQ and Cyclone coverages were 368× and 418×, respectively. The finished genome is a single circular chromosome of 3,397,625 bp, with 53.43% G + C content and 3,116 predicted genes.

## Data Availability

This whole-genome sequencing project has been deposited in DDBJ/GenBank under accession numbers. The data can be found under BioSample accession number SAMD01798706 and BioProject accession number PRJDB40189. The raw data are available from the Sequence Read Archive under accession number DRR1046508 and DRR1046509. The complete genome sequence is available under the accession number AP046527.

## References

[1] Sanford PA, Woolston BM. 2022. Synthetic or natural? metabolic engineering for assimilation and valorization of methanol. Curr Opin Biotechnol 74:171–179. doi:10.1016/j.copbio.2021.12.001 34952430

[2] Motoyama H, Maki K, Anazawa H, Ishino S, Teshiba S. 1994. Cloning and nucleotide sequences of the homoserine dehydrogenase genes (hom) and the threonine synthase genes (thrC) of the gram-negative obligate methylotroph *Methylobacillus glycogenes*. Appl Environ Microbiol 60:111–119. doi:10.1128/aem.60.1.111-119.1994 PMC201277 8117070

[3] Motoyama H, Yano H, Ishino S, Anazawa H, Teshiba S. 1994. Effects of the amplification of the genes coding for the L-threonine biosynthetic enzymes on the L-threonine production from methanol by a gram-negative obligate methylotroph, *Methylobacillus glycogenes*. Appl Microbiol Biotechnol 42:67–72. doi:10.1007/BF00170226 7765821

[4] Chen Y, Chen Y, Shi C, Huang Z, Zhang Y, Li S, Li Y, Ye J, Yu C, Li Z, Zhang X, Wang J, Yang H, Fang L, Chen Q. 2018. SOAPnuke: a MapReduce acceleration-supported software for integrated quality control and preprocessing of high-throughput sequencing data. Gigascience 7:1–6. doi:10.1093/gigascience/gix120 PMC5788068 29220494

[5] Koren S, Walenz BP, Berlin K, Miller JR, Bergman NH, Phillippy AM, Koren S, Walenz BP, Berlin K, Miller JR, et al.. 2017. Canu: scalable and accurate long-read assembly via adaptive *k*-mer weighting and repeat separation. Genome Res 27:722–736. doi:10.1101/gr.215087.116 PMC5411767 28298431

[6] McKenna A, Hanna M, Banks E, Sivachenko A, Cibulskis K, Kernytsky A, Garimella K, Altshuler D, Gabriel S, Daly M, DePristo MA. 2010. The genome analysis toolkit: a MapReduce framework for analyzing next-generation DNA sequencing data. Genome Res 20:1297–1303. doi:10.1101/gr.107524.110 PMC2928508 20644199

[7] Tanizawa Y, Fujisawa T, Nakamura Y. 2018. DFAST: a flexible prokaryotic genome annotation pipeline for faster genome publication. Bioinformatics 34:1037–1039. doi:10.1093/bioinformatics/btx713 PMC5860143 29106469

